# High MICB expression as a biomarker for good prognosis of colorectal cancer

**DOI:** 10.1007/s00432-020-03159-0

**Published:** 2020-04-18

**Authors:** Qingyang Feng, Shanchao Yu, Yihao Mao, Meiling Ji, Ye Wei, Guodong He, Wenju Chang, Dexiang Zhu, Li Ren, Jianmin Xu

**Affiliations:** Department of General Surgery, Zhongshan Hospital, Fudan University, Shanghai, China

**Keywords:** MICB, Colorectal cancer, Prognosis

## Abstract

**Introduction:**

Major histocompatibility complex (MHC) plays an important role in colorectal cancer (CRC) immunity. However, the function of MHC class I chain-related B (MICB) molecule is not very clear. In this study, we explored the prognostic effect of MICB in colorectal cancer.

**Material and methods:**

From 2008-05 to 2012-11, consecutive CRC patients of Zhongshan Hospital, Fudan University were retrospectively enrolled as primary cohort. The inclusion criteria were as follows: receiving primary radical resection, pathologically confirmed colorectal adenocarcinoma, no treatment before surgery, clinicopathological data available. Another cohort of CRC patients were collected from a public dataset GSE39582 of GEO database from 1987 to 2007 in the same criteria for validation. MICB was detected using immunochemistry and evaluated as prognostic biomarker. The cut-off value of MICB expression was calculated using X-tile software.

**Results:**

Finally, 863 patients were enrolled in the primary cohort, and 556 patients were enrolled in the validation cohort. MICB expression was significantly associated with tumor size and primary histological type in primary cohort, and with primary tumor location and distant metastases in validation cohort. The survival analysis showed that patients with high MICB expression had significantly better overall survival in both primary (*P* = 0.002) and validation (*P* = 0.001) cohorts. The multivariate analysis also confirmed that high MICB expression was a significantly independent protective factor for overall survival in both primary (hazard ratio HR = 0.741, 95% CI 0.594–0.924) and validation (HR = 0.699, 95% CI 0.508–0.961) cohorts.

**Conclusion:**

For stage I–IV CRC patients, MICB was confirmed a novel independent prognostic factor. It could help better stratification of CRC prognosis.

**Electronic supplementary material:**

The online version of this article (10.1007/s00432-020-03159-0) contains supplementary material, which is available to authorized users.

## Background

Colorectal cancer (CRC) is the third most frequently encountered cancer among adults (Siegel et al. 2017; Bray et al. 2018) and is the second in terms of mortality. As a highly heterogeneous disease, tumor immunity plays an important role in CRC.

Immune surveillance can eliminate cancer cells in human body. This process is closely related to major histocompatibility complex and its associated molecules. The human major histocompatibility complex (MHC) class I chain-related genes locate in the HLA class I region of chromosome 6 (Stephens 2001). The MHC class I chain-related B molecule, also commonly known as MICB, is one of the ligands of NKG2D receptor. NKG2D receptors exist in NK cells and CD8^+^ T cells, which mediate antitumor response and immune surveillance (Diefenbach et al. 2001). MICB is expressed by the intestinal epithelium and epithelial tumors as well (Groh et al. 1999).

Cancer cells express MICB as the consequence of cellular stress such as genomic damage (Andrade 2018). MICB can tag these cells for elimination. But tumor cells under antibody-dependent cell-mediated cytotoxicity might develop evasive pathways to avoid NK cell attack (Hu et al. 2019).Shedding is a good way for cancer cells to remove or avoid the surface expression of ligands such as MICB with the presence of metalloproteases in the tumor microenvironment (Schmiedel and Mandelboim 2018). Some previous studies reported that MICB was associated with distant metastasis and advanced stages (Kopp et al. 2009), and associated with rejection to tumors in transplanted mice model (Diefenbach et al. 2001). However, there is still a lack of clinical data confirming the prognostic value of MICB.

Here in this study, we detected MICB expression in CRC tissues, and figured out the relationship between MICB and prognosis in a CRC cohort of Zhongshan Hospital, Fudan University. The prognostic benefit was also validated in a public dataset GSE39582 of GEO database.

## Materials and methods

### Study population

Consecutive CRC patients of Zhongshan Hospital, Fudan University from 2008-05 to 2012-11 were retrospectively enrolled in this study as primary cohort. The inclusion criteria were as follows: receiving primary radical resection, pathologically confirmed colorectal adenocarcinoma, no treatment before surgery, clinicopathological data available. CRC cancer stages were determined according to the International Union Against Cancer (UICC)/American Joint Committee on Cancer (AJCC) TNM classification 8th edition. Radical resections of synchronous liver metastases were also permitted.

The public dataset for validation was selected as: (1) transcriptomic data such as microarray data which include MICB were available; (2) the basic clinical and pathological information including detailed TNM stage and overall survival(OS) was available; (3) the size of dataset was larger than 100; (4) the minimum median follow-up time was 36 months. Thus, GSE39582 dataset was selected from Gene Expression Omnibus (GEO) repository (Marisa et al. 2013). CRC patients in GSE39582 dataset between 1987 and 2007 were enrolled as validation cohort in the same criteria as primary cohort. And the data of mRNA expression from dataset GSE39582 were also obtained.

This study was approved by the Clinical Research Ethics Committee of Zhongshan Hospital, Fudan University. Informed consent was acquired from all patients of primary cohort for the acquisition of clinical and pathological information and the use of surgical specimens. Since GSE39582 was a public dataset, approval of the ethics committee and informed consent from the patients was unnecessary.

### Immunohistochemistry

For primary cohort, immunohistochemistry was used to detect the MICB expression. The detailed procedure of the experiment was described as previously reported (Mao 2018). The primary antibody was rabbit anti-human polyclonal MICB (diluted 1:100, ARG56879, Arigo). The secondary antibody was goat anti-rabbit. The MICB intensity of +++ was 3, ++ was 2, + was 1, − was 0. The area score was the percentage of positive cells among all tumor cells multiplied by 100. Finally, the MICB score was MICB intensity multiplied by area score, ranging from 0 to 300.

### Statistical analysis

The statistical analyses were performed using the SPSS 25.0. The association between clinicopathological features and MICB were accessed by Chi-square test or Fisher’s exact test as appropriate. Kaplan–Meier analysis and Log-rank test were performed to evaluate the relationship between MICB expression and OS. Univariate cox regression analyses were performed to identify the independent prognostic factors among clinicopathological features and other information. Those factors with *P* < 0.1 in univariate cox regression analyses were included in the multivariate cox regression analysis. A two-sided *P* < 0.05 was considered statistically significant.

The cut-off values of MICB score were calculated for primary and validation cohorts, respectively, because of the different detection method of MICB expression. And the cut-off values were based on the OS data. To obtain the best prognostic efficacy, X-Tile Software (Yale University, version 3.6.1) was used as previously described (Camp et al. 2004).

## Results

### Patient characteristics

Finally, 863 CRC patients from Zhongshan hospital, Fudan University were enrolled as primary cohort. And 556 patients from dataset GSE39582 were enrolled as validation cohort. The median follow-up time was 60.5 months for primary cohort (IQR = 24.8–91.0) and was 52.0 months (IQR = 27.0–80.0) for validation cohort. 319 (37.0%) patients of primary cohort died by the time of analysis. And 190 (34.2%) patients of GSE39582 died by the time of analysis. Baseline clinical and pathological characteristics of primary and validation cohort are presented in Table 1.

**Table 1:** Baseline clinicopathological characteristics of primary and validation cohorts

Primary cohort			Validation cohort			*P*
Factors	No	%	Factors	No	%	
All patients	863	100.0	All patients	556	100.0	
Age (years)			Age (years)			
≤ 60	415	48.1	≤ 60	157	28.2	** < 0.001**
> 60	448	51.9	> 60	398	71.6	
Unknown	0	0	Unknown	1	0.2	
Gender			Gender			
Male	505	58.5	Male	306	55.0	0.196
Female	358	41.5	Female	250	45.0	
CEA (ng/ml)						
≤ 5	450	52.1				
> 5	413	47.9				
Tumor location			Tumor location			
Right-sided colon	246	28.5	Proximal colon	218	39.2	** < 0.001**
Left-sided colon	226	26.2	Distal colon	338	60.8	
Rectum	391	45.3				
Tumor size (cm)						
≤ 4.0	478	55.4				
> 4.0	385	44.6				
Primary histological type						
Non-mucinous	732	84.8				
Mucinous	131	15.2				
Primary differentiation						
Well/moderate	584	67.7				
Poor/anaplastic	279	32.3				
T stage			T stage			
T1	29	3.4	T1	11	2.0	** < 0.001**
T2	120	13.9	T2	44	7.9	
T3	207	24.0	T3	362	65.1	
T4	507	58.7	T4	119	21.4	
			Unknown	20	3.6	
N stage			N stage			
N0	457	53.0	N0	294	52.9	0.502
N1	264	30.6	N1/N2	242	43.5	
N2	142	16.4	Unknown	20	3.6	
Vascular invasion						
No	761	88.2				
Yes	102	11.8				
Nerve invasion						
No	800	92.7				
Yes	63	7.3				
M stage			M stage			
M0	642	74.4	M0	473	85.1	** < 0.001**
M1	221	25.6	M1	61	11.0	
			Unknown	22	4.0	
TNM stage			TNM stage			
I	114	13.2	I	32	5.8	** < 0.001**
II	282	32.7	II	261	46.9	
III	246	28.5	III	203	36.5	
IV	221	25.6	IV	60	10.8	

Bold values indicate *P* < 0.05

### IHC findings

Representative images of MICB + IHC staining are presented in Supplementary Figure S1. A typical image of 400× high power field is presented in Supplementary Figure S2.

### Definition of cut-off values

The cut-off value was defined as follows. For MICB expression score detected by IHC in primary cohort, 0–135 was defined as low, and 136–300 was defined as high. For MICB expression score detected by mRNA in validation cohort, 3.97–6.58 was defined as low, and 6.59–9.53 was defined as high.

### Association between MICB and clinical characteristics

The relationship between MICB expression and clinicopathological features of primary cohort is presented in Table 2. High MICB expression was significantly associated with non-mucinous histological type (*P* < 0.001) and tumor size ≤ 4.0 cm (*P* = 0.001).

**Table 2:** Relationship between MICB and clinical characteristics of primary cohort

Factors	Primary cohort		
	MICB expression		
	Low (%)	High (%)	*P*
All patients	417	446	
Age (years)			
≤ 60	197 (47.2)	218 (48.9)	0.631
> 60	220 (52.8)	228 (51.1)	
Gender			
Male	240 (57.6)	265 (59.4)	0.579
Female	177 (42.4)	181 (40.6)	
CEA (ng/ml)			
≤ 5	222(53.2)	228 (51.1)	0.534
> 5	195(46.8)	218 (48.9)	
Tumor location			
Right-sided colon	119 (28.5)	127 (28.5)	0.937
Left-sided colon	107 (25.7)	119 (26.7)	
Rectum	191 (45.8)	200 (44.8)	
Tumor size (cm)			
≤ 4.0	227 (54.4)	294 (65.9)	**0.001**
> 4.0	190 (45.6)	152 (34.1)	
Primary histological type			
Non-mucinous	329 (78.9)	403 (90.4)	** < 0.001**
Mucinous	88 (21.1)	43 (9.6)	
Primary differentiation			
Well/moderate	290 (69.5)	294 (65.9)	0.255
Poor/anaplastic	127 (30.5)	152 (34.1)	
T stage			
T1/T2	67 (16.1)	82 (18.4)	0.368
T3/T4	350 (83.9)	364 (81.6)	
N stage			
N0	221 (53.0)	236 (52.9)	0.981
N1/N2	196 (47.0)	210 (47.1)	
Vascular invasion			
No	367 (88.0)	394 (88.3)	0.880
Yes	50 (12.0)	52 (11.7)	
Nerve invasion			
No	388 (93.0)	412 (92.4)	0.706
Yes	29 (7.0)	34 (7.6)	
M stage			
M0	302 (72.4)	340 (76.2)	0.200
M1	115 (27.6)	106 (23.8)	
TNM stage			
I	48 (11.5)	66 (14.8)	0.130
II	145 (34.8)	137 (30.7)	
III	109 (26.1)	137 (30.7)	
IV	115 (27.6)	106 (23.8)	

Bold values indicate *P* < 0.05

The relationship between MICB expression and clinicopathological characteristics of validation cohort is presented in Supplementary Table S1. High MICB expression was significantly associated with tumor located at proximal colon (*P* < 0.001) and M0 stage(*P* = 0.047).

### MICB as prognostic biomarkers

**Fig. 1:**
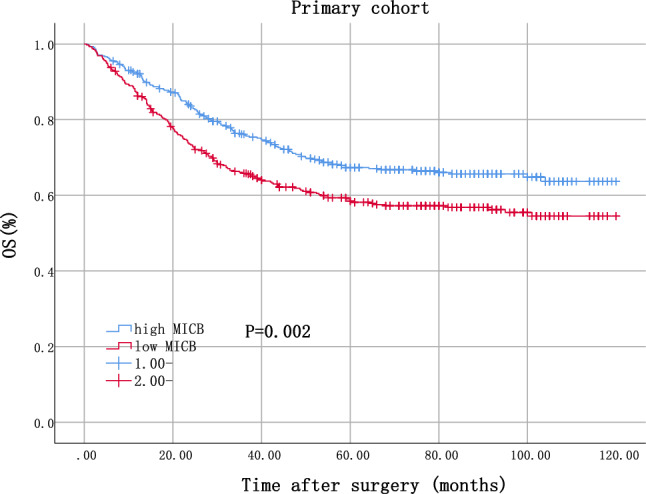
Kaplan–Meier analysis of primary cohort

In primary cohort, patients with high MICB expression had significantly better OS (*P* = 0.002, as shown in Fig. 1). In validation cohort, patient with high MICB expression also had significantly better OS (*P* = 0.001, as shown in Supplementary Figure S3).

For primary cohort, the univariate cox regression analysis showed that preoperative CEA level, tumor location, primary differentiation, T stage, N stage, M stage and MICB expression were significantly associated with OS. In multivariate analyses, MICB expression, preoperative CEA level, N stage and M stage were confirmed as independent prognostic factors for OS (Table 3).

**Table 3:** Cox regression analyses for OS of primary cohort

Factors	Overall survival			
	Univariate analysis		Multivariate analysis	
	HR (95% CI)	*P*	HR (95% CI)	*P*
Age (years)				
≤ 60	1 (reference)	0.644		
> 60	1.053 (0.845–1.312)			
Gender				
Male	1 (reference)	0.062	1 (reference)	0.339
Female	0.805 (0.641–1.011)		0.893 (0.708–1.126)	
CEA (ng/ml)				
≤ 5	1 (reference)	** < 0.001**	1 (reference)	**0.003**
> 5	2.540 (2.017–3.197)		1.441 (1.129–1.840)	
Tumor location				
Right-sided colon	1 (reference)	**0.028**	1 (reference)	0.183
Left-sided colon	1.000 (0.754–1.326)		0.854 (0.640–1.139)	
Rectum	0.737 (0.566–0.958)		0.779 (0.596–1.017)	
Tumor size (cm)				
≤ 4.0	1 (reference)	0.168		
> 4.0	1.167 (0.937–1.455)			
Primary histological type				
Non-mucinous	1 (reference)	0.984		
Mucinous	0.997 (0.731–1.359)			
Primary differentiation				
Well/moderate	1 (reference)	** < 0.001**	1 (reference)	0.098
Poor/anaplastic	1.628 (1.301–2.038)		1.213 (0.965–1.525)	
T stage				
T1/T2	1 (reference)	** < 0.001**	1 (reference)	0.062
T3/T4	3.198 (2.074–4.932)		1.541 (0.978–2.429)	
N stage				
N0	1 (reference)	** < 0.001**	1 (reference)	** < 0.001**
N1/N2	2.775 (2.201–3.499)		1.752 (1.375–2.232)	
M stage				
M0	1 (reference)	** < 0.001**	1 (reference)	** < 0.001**
M1	7.922 (6.318–9.933)		6.029 (4.718–7.702)	
Vascular invasion				
No	1 (reference)	0.819		
Yes	1.041 (0.739–1.466)			
Nerve invasion				
No	1 (reference)	0.234		
Yes	0.755 (0.475–1.200)			
TNM stage				
I	1 (reference)	** < 0.001**		
II	1.771 (0.947–3.315)			
III	3.005 (1.632–5.533)			
IV	16.535 (9.200–29.718)			
MICB				
Low	1 (reference)	**0.002**	1 (reference)	**0.008**
High	0.708 (0.568–0.883)		0.741 (0.594–0.924)	

Bold values indicate *P* < 0.05

For validation cohort, the univariate analysis showed that age, T stage, N stage, M stage and MICB expression were significantly associated with OS. In multivariate analysis, MICB expression, age and M stage were confirmed as independent prognostic factors for OS (Supplementary Table S2).

### Stratified analysis of primary cohort

In primary cohort, stratified analysis was conducted according to TNM stage. For Stage I and II patients, MICB expression was not a significant prognostic factor (*P* = 0.214, Supplementary Figure S4A). For Stage III and IV patients, the survival curves of MICB expression were significant (*P* = 0.001, Supplementary Figure S4B). The cox regression showed that MICB expression was a significant independent prognostic factor for OS (*P* = 0.009, Supplementary Table S3).

**Fig. 2:**
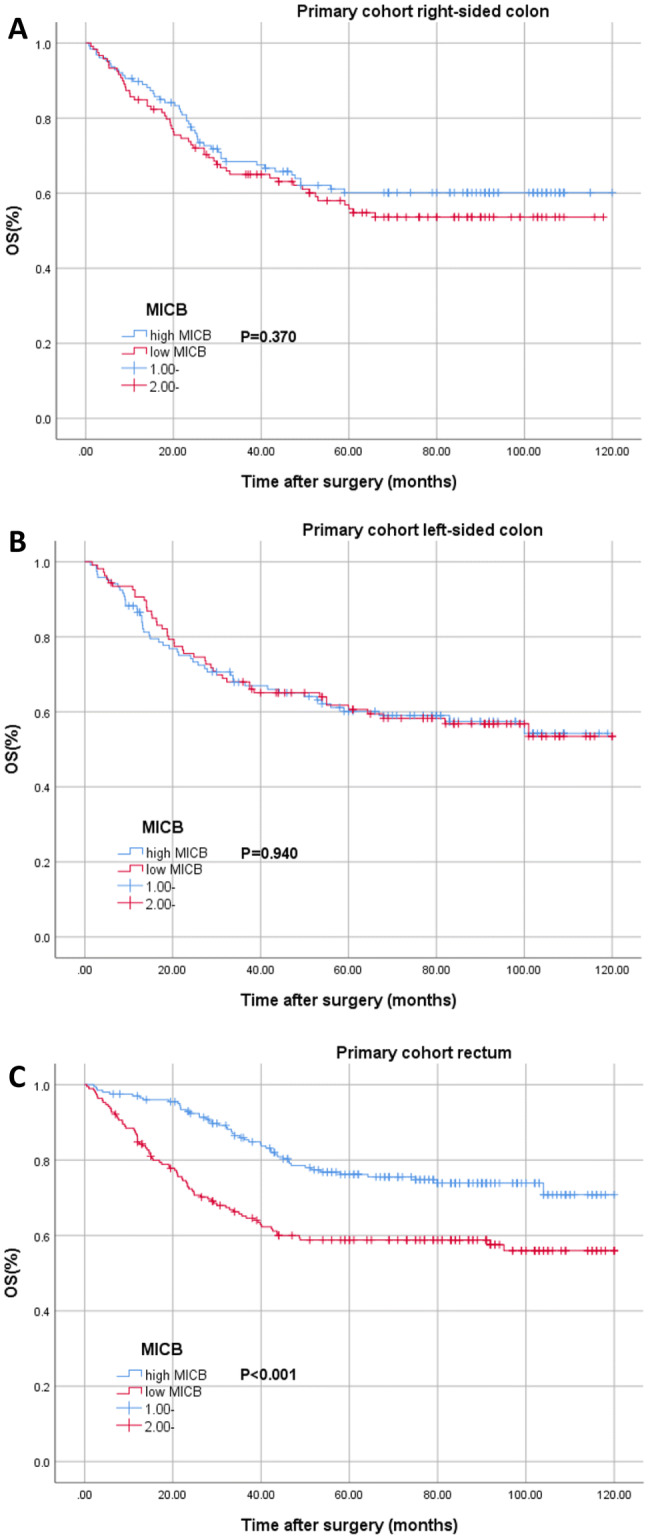
Kaplan–Meier analysis of patients with right-sided colon (**a**), left-sided colon (**b**) and rectal (**c**) cancer from primary cohort

In stratified analysis according to tumor location, for patients with right-sided or left-sided colon cancer, MICB expression was not a significant prognostic factor (Fig. 2a, b). However, for patients with rectal cancer, the survival curves of MICB expression were significant (*P* < 0.001, Fig. 2c). And the cox regression showed that MICB expression was a significantly independent prognostic factor for OS (*P* < 0.001, Table 4).

**Table 4:** Cox regression analyses for OS of patients with rectal cancer from primary cohort

Factors	Overall survival			
	Univariate analysis		Multivariate analysis	
	HR (95% CI)	*P*	HR (95% CI)	*P*
Age (years)				
≤ 60	1 (reference)	0.623		
> 60	1.092 (0.769–1.549)			
Gender				
Male	1 (reference)	0.453		
Female	0.871 (0.607–1.250)			
CEA (ng/ml)				
≤ 5	1 (reference)	** < 0.001**	1 (reference)	**0.003**
> 5	2.346 (1.641–3.354)		1.747 (1.209–2.526)	
Tumor size (cm)				
≤ 4.0	1 (reference)	0.306		
> 4.0	1.203 (0.845–1.714)			
Primary histological type				
Non-mucinous	1 (reference)	0.701		
Mucinous	1.111 (0.648–1.906)			
Primary differentiation				
Well/moderate	1 (reference)	**0.004**	1 (reference)	0.098
Poor/anaplastic	1.667 (1.172–2.371)		1.358 (0.945–1.951)	
T stage				
T1/T2	1 (reference)	** < 0.001**	1 (reference)	0.351
T3/T4	2.564 (1.538–4.275)		1.305 (0.746–2.282)	
N stage				
N0	1 (reference)	** < 0.001**	1 (reference)	**0.013**
N1/N2	2.371 (1.652–3.404)		1.606 (1.104–2.336)	
M stage				
M0	1 (reference)	** < 0.001**	1 (reference)	** < 0.001**
M1	5.494 (3.856–7.828)		4.303 (2.976–6.223)	
Vascular invasion				
No	1 (reference)	0.360		
Yes	1.269 (0.761–2.116)			
Nerve invasion				
No	1 (reference)	0.840		
Yes	0.935 (0.490–1.785)			
TNM stage				
I	1 (reference)	** < 0.001**		
II	1.595 (0.755–3.368)			
III	2.559 (1.272–5.146)			
IV	9.924 (5.058–19.473)			
MICB				
Low	1 (reference)	** < 0.001**	1 (reference)	** < 0.001**
High	0.492 (0.343–0.705)		0.510 (0.356–0.733)	

Bold values indicate *P* < 0.05

## Discussion

In this study, a large cohort of real world was constructed as primary cohort to evaluate the prognostic benefit of MICB in colorectal cancer patients. The positive association between MICB and OS was found in our cohort. And MICB was also identified as a new independent prognostic indicator of OS in CRC patients. Then, the prognostic value of MICB was also validated in GES39582, which is a public GEO cohort.

Stratified analysis indicated that our results are more useful for patients with rectal cancer. This may be related to the rupture of rectal wall caused by feces. As a result, more tumor antigens enter the systemic circulation and stimulate the immune response. Stratified analysis also suggested that stage III and IV patients with high MICB expression had a better prognosis. This may be related to lymph node metastasis, further stimulating the immune response.

The potential mechanism of MICB as a prognostic indicator in CRC patients might be explained by a previous study. Overexpression of microRNA, including miR-17-5p, miR-20a, miR-93, miR-106b, miR-372, miR-373 and miR-520c, resulted in downregulation of MICB expression on the surface of cancer cells and less susceptibility to NKG2D-dependent killing by NK cells (Stern-Ginossar et al. 2008). Eventually, the downregulation of MICB expression enables the tumor to avoid immune recognition which explains the reason of a worse OS in low MICB patients with CRC. Asking for the therapeutic value of MICB, Ferrari de Andrade et al. found that MICB a3 domain-specific antibodies substantially increased the density of the stimulatory MICB ligands on the surface of cancer cells, reduced shed MICB amounts, and induced NK cell attack against cancer cells (Andrade 2018). This suggests that elevating MICB level could be a potential therapy for CRC patients.

However, a couple of limitations of this study must be noticed. First, our study is a retrospective one. To further validate our conclusion, a prospective study with data from multiple centers is necessary, especially for patients with rectal cancer as well as stage III and IV patients. Second, MICB intensity and area score were not detected and determined automatically, resulting in potential artificial errors. Third, the MICB detection method of GSE39582 is different from the method of primary cohort. And the cut-off value of MICB expression was not fully verified.

## Conclusion

In summary, MICB was identified as a new independent prognostic factor for CRC patients. CRC with high MICB expression conferred survival benefit. This could promote the individualized treatment of colorectal cancer.

## Electronic supplementary material

Below is the link to the electronic supplementary material.

Supplementary file1 (DOCX 9939 kb)

## Data Availability

The datasets used and analyzed during the current study are available from the corresponding author on reasonable request.

## Abbreviations

CRC: Colorectal cancer
MHC: Major histocompatibility complex
MICB: MHC class I chain-related B molecule
GEO: Gene Expression Omnibus
OS: Overall survival
IQR: Interquartile range
