# Supplementary material for: High MICB expression as a biomarker for good prognosis of colorectal cancer
Source: J Cancer Res Clin Oncol. 2020 Apr 18;146(6):1405–13. doi: 10.1007/s00432-020-03159-0 (PMC7230058; doi:10.1007/s00432-020-03159-0)
Supplement: Supplementary file 1 — Supplementary file1 (DOCX 9939 kb) [file 432_2020_3159_MOESM1_ESM.docx]

Figure S1 Representative images of MICB+ staining


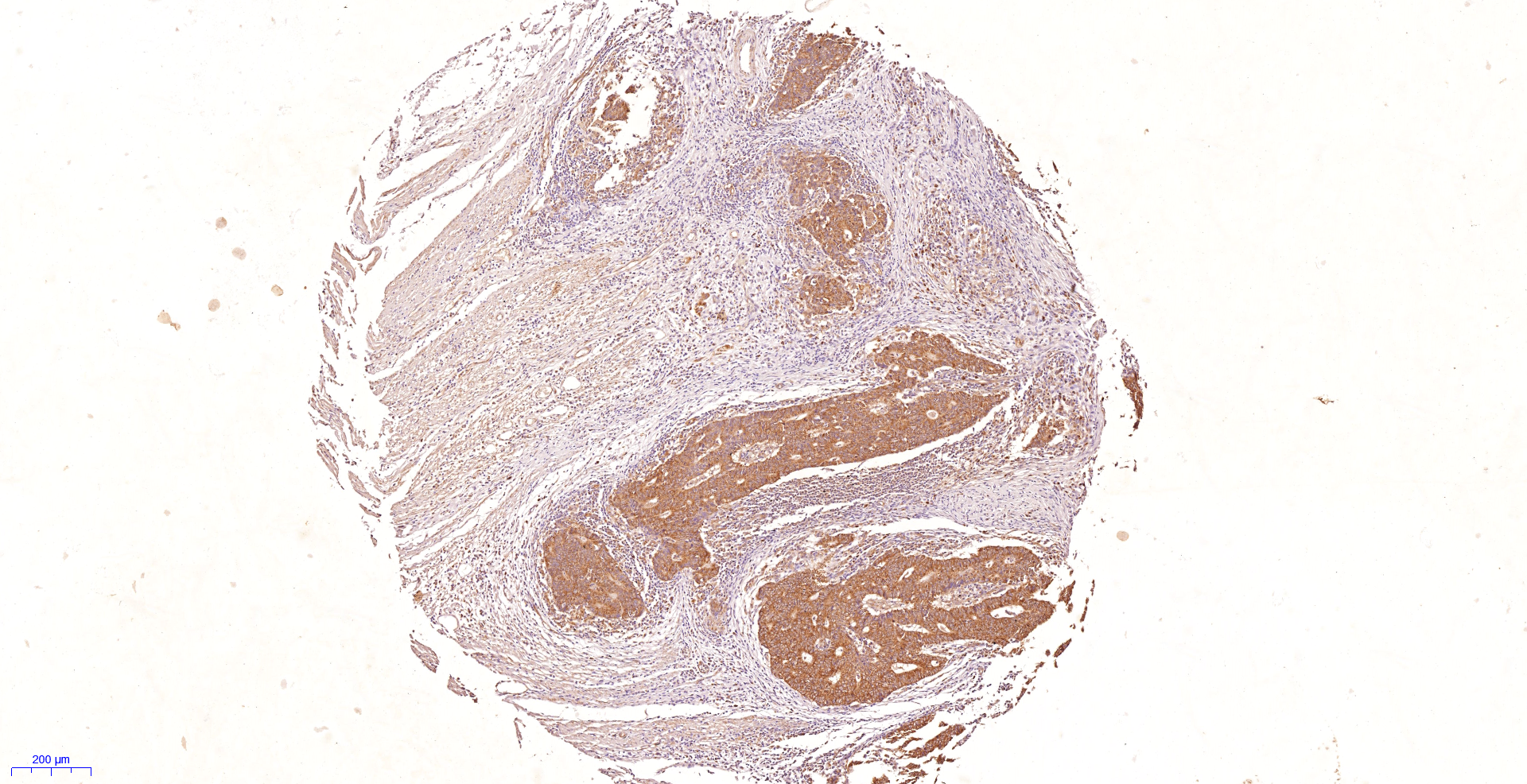


**+++**


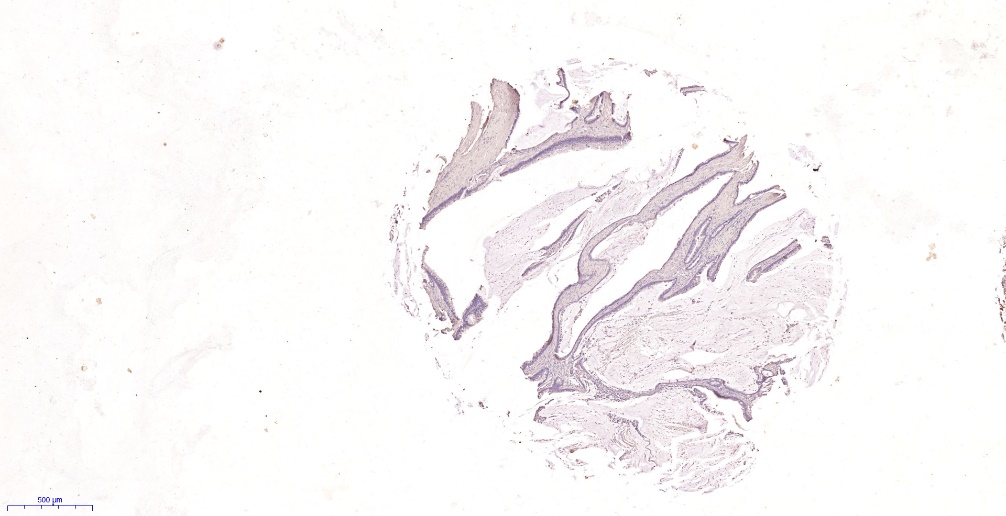


**-**


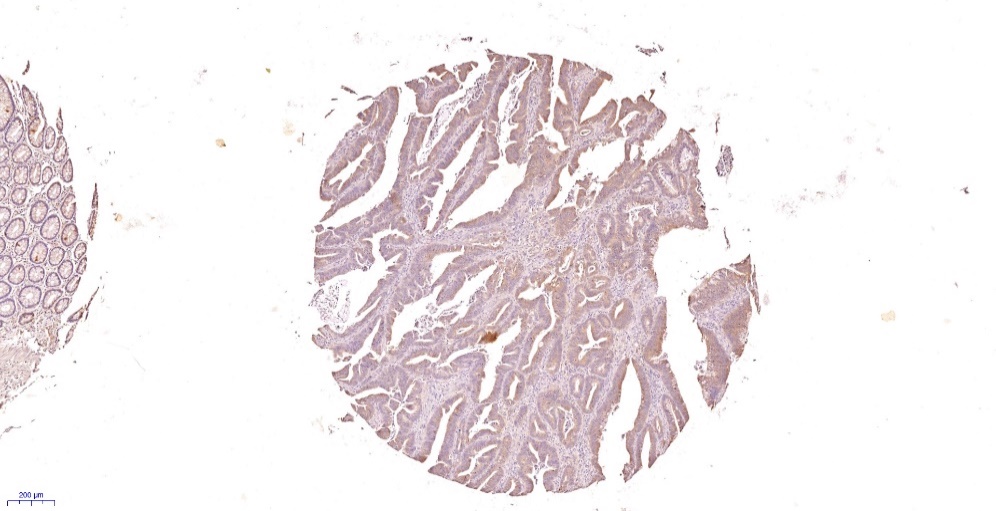


**+**


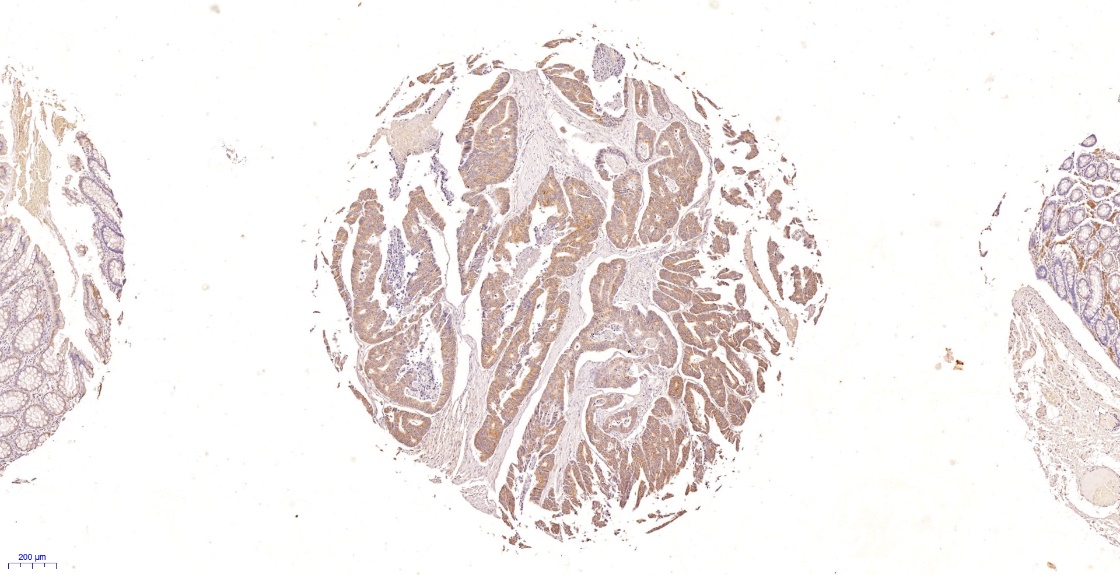


**++**

**A**

**B**

**C**

**D**

Figure S2 A typical image of 400x high power field of MICB+ staining


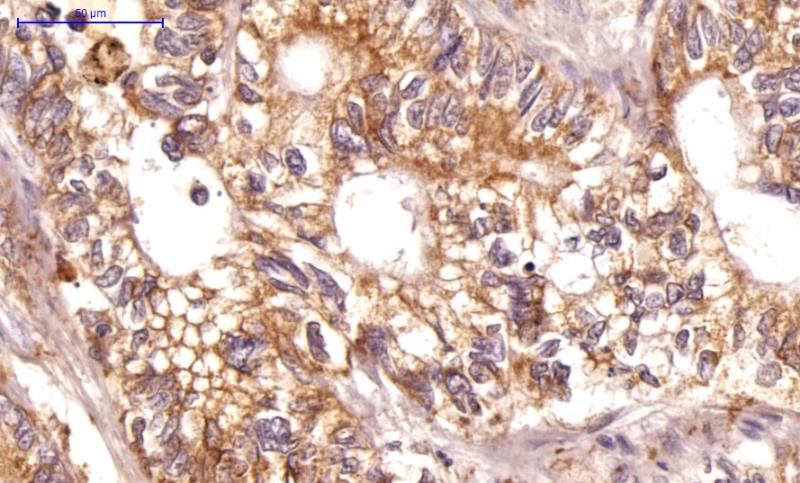


Table S1 Relationship between MICB and clinical characteristics of validation cohort

| **validation cohort** | | | | |
| --- | --- | --- | --- | --- |
|  |  | **MICB** | | |
| Factors | No (%) | Low (%) | High (%) | *P* |
| All patients | 556 | 342 | 214 |  |
| Age (years) |  |  |  | 0.242 |
| ≤60 | 157 (28.2) | 91 (26.6) | 66 (30.8) |  |
| >60 | 398 (71.6) | 251 (73.4) | 147 (68.7) |  |
| Unknown | 1 (0.2) | 0 (0.2) | 1 (0.5) |  |
| Gender |  |  |  | 0.508 |
| Male | 306 (55.0) | 192 (56.1) | 114 (53.3) |  |
| Female | 250 (45.0) | 150 (43.9) | 100 (46.7) |  |
| Localization |  |  |  | **<0.001** |
| Proximal colon | 218 (39.2) | 112 (32.7) | 106 (49.5) |  |
| Distal colon | 338 (60.8) | 230 (67.3) | 108 (50.5) |  |
| T stage |  |  |  | 0.070 |
| T1/T2 | 55 (9.9) | 31 (9.1) | 24 (11.2) |  |
| T3/T4 | 481 (86.5) | 294 (86.0) | 187 (87.4) |  |
| Unknown | 20 (3.6) | 17 (5.0) | 3 (1.4) |  |
| N stage |  |  |  | 0.075 |
| N0 | 294 (52.9) | 175 (51.2) | 119 (55.6) |  |
| N1/N2 | 242 (43.5) | 150 (43.9) | 92 (43.0) |  |
| Unknown | 20 (3.6) | 17 (5.0) | 3 (1.4) |  |
| M stage |  |  |  | **0.047** |
| M0 | 473 (85.1) | 281 (82.2) | 192 (89.7) |  |
| M1 | 61 (11.0) | 44 (12.9) | 17 (7.9) |  |
| Unknown | 22 (4.0) | 17 (5.0) | 5 (2.3) |  |
| TNM stage |  |  |  | 0.345 |
| I | 32 (5.8) | 18 (5.3) | 14 (6.5) |  |
| II | 261 (46.9) | 156 (45.6) | 105 (49.1) |  |
| III | 203 (36.5) | 125 (36.5) | 78 (36.4) |  |
| IV | 60 (10.8) | 43 (12.6) | 17 (7.9) |  |

Figure S3 Kaplan-Meier analysis of validation cohort


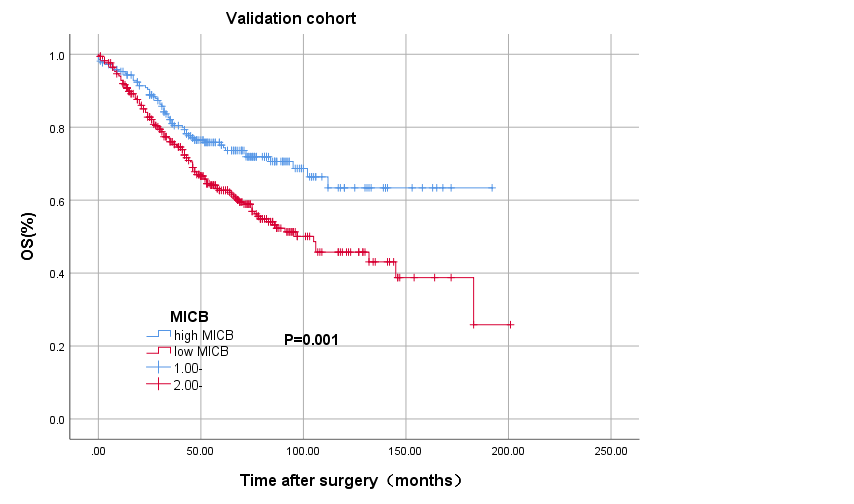


Table S2. Univariate and multivariate Cox regression analysis for overall survival in validation cohort

|  | **Overall survival** | | | |
| --- | --- | --- | --- | --- |
|  | **Univariate analysis** | | **Multivariate analysis** | |
| Factors | HR (95% CI) | *P* | HR (95% CI) | *P* |
| Age (years) |  | **0.034** |  | **0.007** |
| ≤60 | 1 (reference) |  | 1 (reference) |  |
| >60 | 1.450 (1.029 – 2.043) |  | 1.624 (1.145 – 2.304) |  |
| Gender |  | 0.076 |  | 0.083 |
| Male | 1 (reference) |  | 1 (reference) |  |
| Female | 0.769 (0.575 – 1.028) |  | 0.771 (0.575 – 1.035) |  |
| Localization |  | 0.600 |  |  |
| Right-sided colon | 1 (reference) |  |  |  |
| Left-sided colon | 0.925 (0.690 – 1.239) |  |  |  |
| T stage |  | **0.044** |  | 0.153 |
| T1/T2 | 1 (reference) |  | 1 (reference) |  |
| T3/T4 | 1.990 (1.017 – 3.891) |  | 1.635 (0.833 – 3.208) |  |
| N stage |  | **0.001** |  | 0.061 |
| N0 | 1 (reference) |  | 1 (reference) |  |
| N1/N2 | 1.663 (1.237 – 2.234) |  | 1.351 (0.986 – 1.850) |  |
| M stage |  | **<0.001** |  | **<0.001** |
| M0 | 1 (reference) |  | 1 (reference) |  |
| M1 | 5.171 (3.608 – 7.410) |  | 4.603 (3.136 – 6.758) |  |
| TNM stage |  | **<0.001** |  |  |
| I | 1 (reference) |  |  |  |
| II | 1.787 (0.723 – 4.417) |  |  |  |
| III | 2.198 (0.886 – 5.454) |  |  |  |
| IV | 8.901 (3.502 – 22.623) |  |  |  |
| MICB |  | **0.001** |  | **0.028** |
| Low | 1 (reference) |  | 1 (reference) |  |
| High | 0.594 (0.434 – 0.811) |  | 0.699 (0.508 – 0.961) |  |

Figure S4 Kaplan-Meier analysis of stage I and II patients from primary cohort(A) and Kaplan-Meier analysis of stage III and IV patients from primary cohort(B)


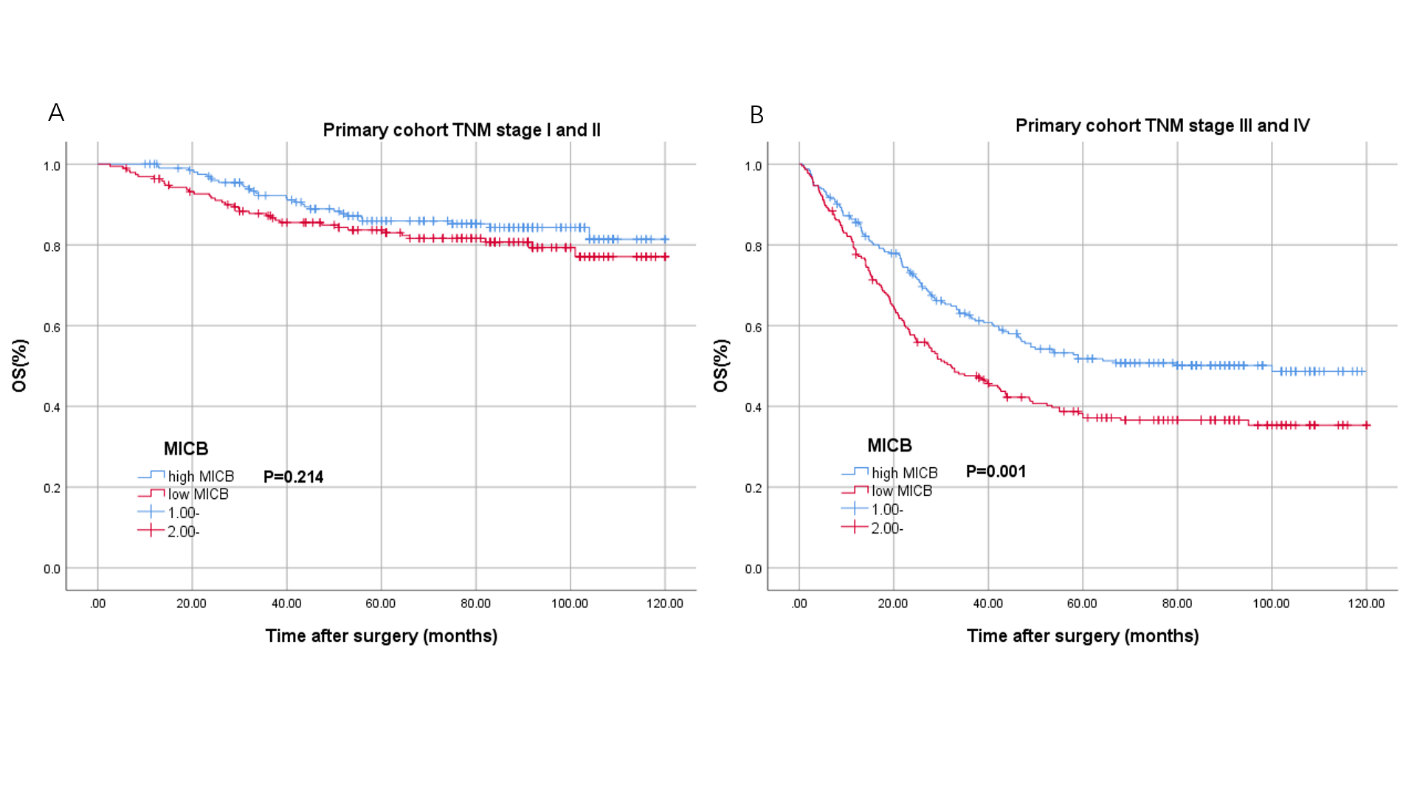


Table S3 Cox regression analyses for OS of stage III and IV patients from primary cohort

|  | **Overall survival** | | | |
| --- | --- | --- | --- | --- |
|  | **Univariate analysis** | | **Multivariate analysis** | |
| Factors | HR (95% CI) | *P* | HR (95% CI) | *P* |
| Age (years) |  | 0.416 |  |  |
| ≤60 | 1 (reference) |  |  |  |
| >60 | 1.097 (0.858 –1.403) |  |  |  |
| Gender |  | **0.032** |  | 0.429 |
| Male | 1 (reference) |  | 1 (reference) |  |
| Female | 0.756 (0.585 –0.976) |  | 0.899（0.691-1.170） |  |
| CEA (ng/ml) |  | **<0.001** |  | **0.004** |
| ≤ 5 | 1 (reference) |  | 1 (reference) |  |
| > 5 | 2.271(1.723-2.993) |  | 1.522 (1.144-2.024) |  |
| Tumor location |  | **0.007** |  | 0.156 |
| Right-sided colon | 1 (reference) |  | 1 (reference) |  |
| Left-sided colon | 0.958(0.703-1.307) |  | 0.951 (0.685-1.321) |  |
| Rectum | 0.652(0.485-0.878) |  | 0.759 (0.560-1.029) |  |
| Tumor size |  | **0.032** |  | **<0.001** |
| ≤4.0cm | 1 (reference) |  | 1 (reference) |  |
| >4.0cm | 1.311(1.024-1.678) |  | 1.562 (1.215-2.006) |  |
| Primary histological type |  | 0.563 |  |  |
| Non-mucinous | 1 (reference) |  |  |  |
| Mucinous | 1.111(0.777-1.588) |  |  |  |
| Primary differentiation |  | 0.132 |  |  |
| Well/moderate | 1 (reference) |  |  |  |
| Poor/anaplastic | 1.210(0.944-1.551) |  |  |  |
| T stage |  | **0.016** |  | 0.461 |
| T1/T2 | 1 (reference) |  | 1 (reference) |  |
| T3/T4 | 2.179 (1.158 – 4.101) |  | 1.278(0.665-2.458) |  |
| N stage |  | **0.003** |  | **0.003** |
| N0 | 1 (reference) |  | 1 (reference) |  |
| N1/N2 | 0.616 (0.446 –0.850) |  | 1.664 (1.184-2.338) |  |
| M stage |  | **<0.001** |  | **<0.001** |
| M0 | 1 (reference) |  | 1 (reference) |  |
| M1 | 5.349 (4.060 – 7.048) |  | 5.757 (4.269 – 7.763) |  |
| Vascular invasion |  | 0.624 |  |  |
| No | 1 (reference) |  |  |  |
| Yes | 1.102(0.748-1.622) |  |  |  |
| Nerve invasion |  | 0.441 |  |  |
| No | 1 (reference) |  |  |  |
| Yes | 0.824(0.504-1.348) |  |  |  |
| MICB |  | **0.001** |  | **0.009** |
| Low | 1 (reference) |  | 1 (reference) |  |
| High | 0.666 (0.520 –0.853) |  | 0.716(0.558-0.919) |  |
